# A PCR-Based Method for RNA Probes and Applications in Neuroscience

**DOI:** 10.3389/fnins.2018.00266

**Published:** 2018-05-02

**Authors:** Ruifang Hua, Shanshan Yu, Mugen Liu, Haohong Li

**Affiliations:** ^1^Ministry of Education Key Laboratory for Biomedical Photonics, Huazhong University of Science and Technology, Wuhan, China; ^2^Department of Biomedical Engineering, School of Life Science and Technology, Huazhong University of Science and Technology, Wuhan, China; ^3^Henan Key Laboratory of Immunology and Targeted Therapy, Henan Collaborative Innovation Center of Molecular Diagnosis and Laboratory Medicine, School of Laboratory Medicine, Xinxiang Medical University, Xinxiang, China; ^4^Department of Genetics and Developmental Biology, College of Life Science and Technology, Huazhong University of Science and Technology, Wuhan, China

**Keywords:** *in situ* hybridization, RNA probe, PCR, transgenic mice, immuno-fluorescence, retrograde tracing

## Abstract

*In situ* hybridization (ISH) is a powerful technique that is used to detect the localization of specific nucleic acid sequences for understanding the organization, regulation, and function of genes. However, in most cases, RNA probes are obtained by *in vitro* transcription from plasmids containing specific promoter elements and mRNA-specific cDNA. Probes originating from plasmid vectors are time-consuming and not suitable for the rapid gene mapping. Here, we introduce a simplified method to prepare digoxigenin (DIG)-labeled non-radioactive RNA probes based on polymerase chain reaction (PCR) amplification and applications in free-floating mouse brain sections. Employing a transgenic reporter line, we investigate the expression of the somatostatin (SST) mRNA in the adult mouse brain. The method can be applied to identify the colocalization of SST mRNA and proteins including corticotrophin-releasing hormone (CRH) and protein kinase C delta type (PKC-δ) using double immunofluorescence, which is useful for understanding the organization of complex brain nuclei. Moreover, the method can also be incorporated with retrograde tracing to visualize the functional connection in the neural circuitry. Briefly, the PCR-based method for non-radioactive RNA probes is a useful tool that can be substantially utilized in neuroscience studies.

## Introduction

*In situ* hybridization (ISH) is a powerful technique, which identifies the localization of specific genes to understand the structure and function of RNAs. In the past decades, this technique has been developed for various types and labeling methods (Yang et al., 1999; Basyuk et al., 2000; Plenz, 2003). Initially, radioactive ISH was used to detect ribosomal DNA sequences due to its high sensitivity, however the development of which is limited by the existence of the harmful radioactive nucleotides (Gall and Pardue, 1969). Recent studies have shown that ISH signals using non-radioactive digoxigenin (DIG)-labeled probes and chromogenic detection methods provide satisfactory results at the single-cell level (Kessler et al., 1990; Nivet et al., 1995; Gandrillon et al., 1996; Klok et al., 2000).

ISH is commonly used in many laboratories, together with multiple anatomical techniques. The combinations of ISH with immunohistochemistry (IHC) and neuronal tracing have been developed to identify the co-localization of multiple genes and neural circuits in neuroscience (Le Moine, 2003; Nehmé et al., 2011; Bonn et al., 2012; Kudo et al., 2012; Parks et al., 2014). All the above mentioned strategies are useful to identify distinct neuronal populations in brain regions of interest or following various stimulations (Zhu et al., 2007; Sonomura et al., 2008). Traditionally, ISH and immunohistochemistry have been used as standards for detecting the specificity of genetic targeting. IHC is more popular for researchers to label the proteins due to its simplified procedures, while ISH identifies the site or cell in which a given protein can potentially be synthesized (Newton et al., 2002). ISH has played an increasingly important role in a variety of studies including analysis of Cre knockin mouse line, medical diagnosis, and gene mapping.

The widespread protocols for the preparation of RNA probes are based on plasmid amplification (Fujimori et al., 1997; Jin and Lloyd, 1997; Newton et al., 2002; Nehmé et al., 2011; Bonn et al., 2012), which are time-consuming and not suitable for rapid assessment of gene expression. Recently, researchers have reported a fast and efficient polymerase chain reaction (PCR) method to prepare RNA probes and have been successfully applied it in liver tissues (Ghafoory et al., 2012).

In this study, we optimized the protocol for the preparation of DIG-labeled RNA probe and also applied it into the free-floating mouse brain sections. The method was based on a two-step PCR amplification. The DIG-labeled probes were *in vitro* transcribed from PCR products, which had high efficiency in current studies. In the applications, we primarily chose routine techniques to validate the practicability. Firstly, using transgenic reporter mice, we revealed the expression of the somatostatin (SST) gene in the adult mouse brain, which could contribute to understanding the heterogeneity in the neurogenic process. Next, we characterized the distribution of SST mRNA and proteins including corticotrophin-releasing hormone (CRH) and protein kinase C delta type (PKC-δ) in specific brain regions. Finally, the functional connection in the neural circuitry was visualized by incorporating retrograde tracing. Illustrative examples show that the PCR-based method for RNA probes is applicable in neuroscience.

## Materials and methods

### Animals

Mice (8–12 weeks of age) were group-housed under a 12-h light-dark cycle (7:00–19:00 light) and bred in the animal facilities at Wuhan National Laboratory for Optoelectronics. The following mouse lines were used in the current study: C57BL/6, SST-Ai3, and CRH-Ai3. All experimental procedures involving animals were approved by the Hubei Provincial Animal Care and Use Committee and were in accordance with the experimental guidelines of the Animal Experimentation Ethics Committee of Huazhong University of Science and Technology, China.

### Surgery

Mice were anesthetized with a mixture of 2% chloral hydrate and 10% urethane diluted in 0.9% NaCl and placed in a stereotaxic injection frame (Narishige Scientific Instrument Laboratory, Japan). For retrograde tracing, 0.3 μl CTB−555 (C34776, Invitrogen, USA, 2 μg/μl in diluted 0.01 M PBS) was delivered by craniotomy into the substantia innominata (SI) with high pressure through a glass micropipette (PMI-100 pressure microinjector, Dagan Ltd.). The stereotaxic coordinates of the SI were AP−0.5 mm; ML ± 1.78 mm; and DV−4.75 mm. The mice were allowed 3 days for sufficient retrograde transport.

### RNA isolation

During the experiments, the solutions were treated with 0.1% diethyl pyrocarbonate (DEPC, 40718, Sigma, USA) and then sterilized. All manipulations were performed on ice and centrifuged at 4°C. RNA was isolated from C57BL/6 mouse brain tissue using TRIzolTM reagent (15596026, Invitrogen, USA). The brain tissue was extracted in 1 min.

The brain tissue was placed in a 1-ml grinder and 1 ml TRIzolTM reagent was added. The homogenized solution was transferred to 1.5-ml RNase-free tubes and then centrifuged at 12,000 × g for 5 min. The supernatant was removed and added with 200 μl chloroform, followed by centrifugation at 12,000 × g for 15 min. Approximately 500 μl of the supernatant was added to 500 μl isopropyl alcohol in a centrifuge tube. After a 10-min reaction time, the tube was centrifuged at 12,000 × g for 10 min. The total RNA precipitate was rinsed with fresh 75% ethanol, followed by centrifugation at 7,500 × g for 5 min. Twenty microliters of autoclaved DEPC–H_2_O was then added. The total RNA was determined with a UV spectrophotometer and gel electrophoresis. The RNA was stored at −80°C for use in reverse transcriptase PCR.

### cDNA synthesis

#### The synthesis of first-strand cDNA

The total reaction volume of 12 μl included 1–5 ng total RNA, 1 μl oligo-dT15 primers (#6110, Takara, Japan), 1 μl 10 nM dNTP mix, and double-distilled H_2_O(ddH_2_O), which was reacted at 65°C for 5 min. Then, 1 μl RNase inhibitor, 3 μl 0.1 M DTT (#1305658, Invitrogen, USA), and 4 μl 5 × first-strand buffer were added and the reaction volume was heated at 37°C for 2 min.

#### The synthesis of double-stranded cDNA

Moloney murine leukemia virus (M–MLV) reverse transcriptase (#28025013, 200 U/μl, Invitrogen, USA) was added (1 μl), and the solution was reacted at 37°C for 2 min. The products were stored at −20°C.

### Primer design

The primers were designed using programs from the U.S. National Center for Biotechnology Information (NCBI) so that they had similar melting temperatures (Tm) and a balanced G/C content, with the avoidance of self-complementarity and any hairpin structure (Ye et al., 2012). Taking into account the specific sequences containing both the RNA polymerase promoter and target DNA sequences, we designed two pairs of primers. The pairs of primer were obtained using NCBI's Primer Blast program or from the Allen Brain Atlas, and these were used to generate RNA probes for SST (391 nt corresponding to nucleotide 138–528, NM_009215.1), NPSR1 (741 nt corresponding to nucleotide 1897–2637, NM_175678.2), and CCKBR (743 nt corresponding to nucleotide 1048–1790, NM_007627.5) (Table 1). Using T7 RNA polymerase, digoxigenin (DIG)-labeled antisense RNA probes were synthesized by *in vitro* transcription.

**Table 1:** Primer pairs used in PCR preparing for ISH probes.

**Primer**	**Sequence (5′->3′)**	**Product (nt)**
SST		391
Forword	CTCTGCATCGTCCTGGCTT	
Rerverse	GGGGCCAGGAGTTAAGGAAG	
EX+T7+Reverse	CAGTGAATTGTAATACGACTCACTATAGGGAGAGGGGCCAGGAGTTAAGGAAG	
NPSR1		741
Forword	CCAGAATGAGAGCTGAGACCTT	
Rerverse	TCTGCCTAAAGAGATGCTTTCC	
EX+T7+Reverse	CAGTGAATTGTAATACGACTCACTATAGGGAGA TCTGCCTAAAGAGATGCTTTCC	
CCKBR		743
Forword	GATGGTGATAATGACAGCGAGA	
Rerverse	ACTGCTTTCTGTGTGTAGGGGT	
EX+T7+Reverse	CAGTGAATTGTAATACGACTCACTATAGGGAGA ACTGCTTTCTGTGTGTAGGGGT	

*The first pair of primers contained the forward and reverse primer, used in the first PCR. The second pair of primers containing forward and EX+T7+reverse primer was used in the second PCR. EX, extra nucleotides; T7, T7 RNA polymerase promoter. Genebank accession no.: SST, somatostatin, NM_009215.1 (refer to https://www.ncbi.nlm.nih.gov/nuccore/6678034); NPSR1, neuropeptide S receptor 1, NM_175678.2 (refer to http://mouse.brain-map.org/experiment/show/70560288); CCKBR, cholecysto-kinin B receptor, NM_007627.5 (refer to http://mouse.brain-map.org/experiment/show/70560342)*.

### Template synthesis using polymerase chain reaction (PCR)

Two-step PCR amplification ensured high fidelity in the synthesis of special DNA sequences and the T7 RNA polymerase promoter.

#### First PCR

The first primer pair was used to obtain the specific complementary DNA sequences from cDNA. The total reaction volume of 50 μl contained 25 μl 2 × ES-Taq MasterMix (CW0690M, Cwbio, China), 2 μl 10 mM upstream primer and 2 μl 10 mM downstream primer, 1 μl cDNA and 20 μl ddH_2_O. Amplification was performed for 3 min at 95°C, followed by 35 cycles, with each consisting of 30 s at 95°C, 30 s at 60°C, and 30 s at 72°C; with a final extension step of 10 min at 72°C. Products were isolated by 1% agarose gel and separated from the gel with a DNA extraction kit (B518131, Sangon Biotech, China).

#### Secondary PCR

The secondary PCR amplification was performed using a larger volume (e.g., 300–400 μl) in a parallel 50-μl reaction system. The amplifications and extraction were performed as the first PCR. Purified PCR products were used as the template for *in vitro* transcription.

### DIG-labeled cRNA probe synthesis

The RNase-free requirements are similar to those used during the RNA isolation procedure. For *in vitro* transcription, the reaction solution with a final volume of 10 μl was prepared with the following components: 2–5 ng purified PCR fragments (template DNA), 1 μl DIG-RNA-labeling mixture (#11175025910, Roche, Germany), 0.25 μl RNase inhibitor (EO0382, Thermo Scientific, USA), 1 μl 0.1 M DTT (#1305658, Invitrogen, USA), and 1 μl T7 RNA polymerase (P2075, Promega, USA). The reaction was incubated at 37°C for 2 h.

Extra template DNA was removed by digesting the products with 2 U RNAse-free DNAse I (M6101, Promega, USA) at 37°C for 30 min. The RNA probe was precipitated by adding 50 μl ethanol and cooling at −20°C for 6 h. The RNA probe was isolated and stored at −80°C in 30 μl DEPC-H_2_O to inactive before use.

### *In situ* hybridization

#### Preparation of brain sections

In this study, 8–12-week-old C57/BL mice were used. The mice were anesthetized with a mixture of 2% chloral hydrate and 10% urethane, followed by an infusion with 0.01 M PBS and 4% paraformaldehyde (PFA, #16005, Sigma, USA). Next, the brains were harvested, post-fixed in 4°C for 6–8 h, and then cut using a vibratome (Leica VT1200S, Leica Microsystems, Germany) into 40-μm-thick sections. The brain sections were placed in a 24-well RNase-free plate (#430828, Corning, Mexico).

#### Hybridization

The RNase-free requirements were maintained up to post-hybridization steps. The sections were washed 3 times with 1 × PBST (0.01 M PBS containing 0.1% Tween-20, P1379, Sigma, USA) for 10 min each time. The tissue sections were given a penetrating treatment with 2 μg/ml proteinase K (diluted in 1 × PBST, AR0056, Bosterbio, USA) solution for 20 min in room temperature (RT), followed by post-fixing with 4% PFA for 10 min. Then the sections were washed 3 times for 10 min each time with 1 × PBST and transferred to 2-ml RNase-free round-bottom tubes.

Prehybridization was performed by incubating the sections at 50°C for 2 h. The prehybridization solution was prepared as follows (for a final volume of 50 ml): 25 ml of deionized formamide (F9037, Sigma, USA), 12.5 ml of 20 × SSC, 250 μl of 20% Tween-20, and 12.25 ml of DEPC-H_2_O (to inactive RNase enzymes).

For hybridization, a hybridization solution was prepared by adding 10 μl heparin (100 mg/ml, H3149, Sigma, USA) and 10 mg yeast tRNA (#10109495001, Roche, Germany) to 20 ml prehybridization solution. The hybridization probe was prepared by adding the RNA probe to the hybridization solution; the final concentration was 2–5 μg/ml. The probe was denatured at 80°C for 10 min and then immediately chilled on ice for 5 min. The sections were incubated at 50°C for 16–20 h.

#### Post-hybridization

The probes were retrieved and stored at −20°C, which can be reused up to 4 times. The sections were washed with 5 × SSCT−50% formamide (v/v) at room temperature (RT) for 5 min. Next, the sections were washed with 2 × SSCT−50% formamide (v/v) at 50°C for 1 h. Then, the sections were washed twice at RT, 15 min each, with 2 × SSCT (2 × SSC containing 0.1% Tween-20). The sections were incubated with 20 μg/ml RNase A (R1253, Thermo Scientific, USA) buffer at RT for 30 min, and then they were washed with 2 × SSCT for 10 min. The sections were incubated with 2 × SSCT−50% formamide (v/v) at 50°C for 1 h and then consecutively washed with 2 × SSCT, 0.2 × SSCT, and 1 × PBST for 15 min each time.

The sections were incubated with blocking buffer (1 × PBST containing 10% normal goat serum (v/v) and 0.2% bovine serum albumin [BSA, B2064, Sigma, USA) (w/v)] at RT for 90 min. Then the sections were incubated with AP-conjugated anti-DIG antibody overnight at 4°C (1093274, Roche, Germany, 1:500 diluted in the blocking buffer).

#### Coloration

The sections were washed 3 times in 1 × PBST, 30 min each time. For a higher signal-to-noise ratio, the washing time can be extended. Then, the sections were washed with fresh AP buffer containing 0.1 M Tris-HCl (pH 9.5), 0.05 M MgCl2, 0.1 M NaCl, and 20 μM levamisole hydrochloride (#31742, Sigma, USA) diluted in double distilled H_2_O. The hybridization signals were detected with nitroblue tetrazolium/(5-bromo-4-chloro-3-indolyl-phosphate) (NBT/BCIP, 11681451001, Roche, Germany, 1:200 diluted in AP buffer) in a light-resistant environment for 30 min-4 h.

### Immunofluorescence

For immunofluorescent staining, brain sections were washed with 0.01 M PBS and incubated with blocking buffer (10% w/v normal goat serum and 0.3% v/v Triton X-100 in 0.01 M PBS) for 90 min at room temperature. After blocking, sections were incubated overnight or 48 h at 4°C with the primary antibodies. The primary antibodies used were rabbit antibody against GFP (ab290, Abcam, 1:600), mouse antibody against PKC-δ (#610397, BD, 1:800), and goat antibody against CTB (#703, List, 1:20,000), which were all diluted in blocking buffer. The sections were washed 3 times with 0.01 M PBS for 10 min each time, and then incubated with secondary antibodies for 2 h at room temperature. The fluorophore-conjugated secondary antibodies used were Alexa Fluor 488-conjugated anti-rabbit IgG antibody (A11008, Invitrogen, 1:1,000), Alexa Fluor 594-conjugated anti-mouse IgG antibody (A11032, Invitrogen, 1:1,000), and Alexa Fluor 594-conjugated anti-goat IgG antibody (A11080, Invitrogen, 1:1,000), which were all diluted in PBS. The secondary antibodies were washed 3 times in 0.01 M PBS, and tissue sections were then mounted onto coverslips and covered with 50% glycerinum (v/v) mixed with diamidino-phenylindole (DAPI, #10236276001, Roche, 5 μg/ml).

### Histological analysis

For investigation of ISH-positive signals, the brain sections were mounted to the slides and covered with 50% glycerol. Images were captured with a Nikon Ni-E biological microscope. For immunofluorescent staining, the images were captured with a Zeiss LSM710 confocal microscope and analyzed with Image J software.

## Results

The protocol illustrates a simplified method for the preparation of RNA probes and applications combined with immunofluorescent staining and retrograde tracing in the central nervous system (Supplementary Figure 1).

### Optimized method to prepare DIG-labeled RNA probe

**Figure 1:**
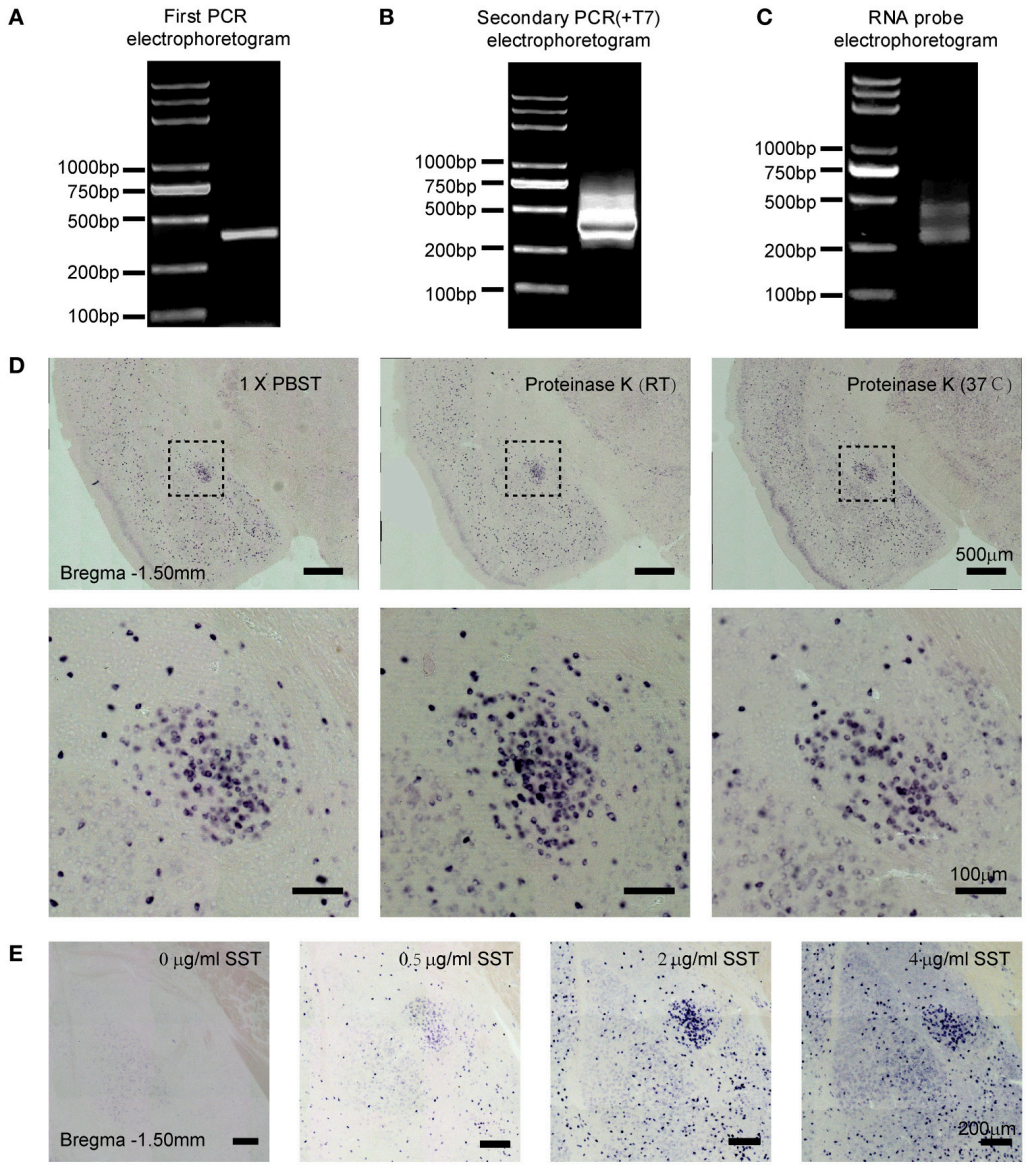
Preparation and visualization of SST probe in mouse brain sections. Two-step polymerase chain reaction (PCR) amplifications were performed and PCR products were examined by agarose gel electrophoresis. **(A)** Products of SST from the first PCR. **(B)** Products of SST containing the T7 promoter from the second PCR. **(C)** *In vitro*-transcribed RNA probe of SST. **(D)** Staining for SST mRNA expression revealed the effect of tissue permeability on signal intensity. The left images show treatment with 1 × PBST (1% Tween-20 in 0.01 M PBS) for 20 min at room temperature (RT); the middle images show treatment with 2 μg/ml proteinase K at RT; and the right images show treatment with 2 μg/ml proteinase K at 37°C. The upper images were captured using 10 × magnification (scale bar = 500 μm), and the bottom images were captured using 20 × magnification (scale bar = 100 μm). **(E)** Sections were stained with different concentrations of the SST probe at 0, 0.5, 2, and 4 μg/ml, respectively. Images were captured using 10 × magnification (scale bar = 200 μm). SST, somatostatin.

In this process, the quality of PCR products is crucial for the specificity of RNA probe. We designed two pairs of somatostatin (SST) primers. The primary amplified products of PCR containing the specific DNA sequences were synthesized from cDNA. The purified PCR products of SST (391 bp) were verified by gel electrophoresis (Figure 1A). For the secondary PCR amplification, T7 promoter sequences were added before the target DNA sequences. It is necessary to determine the purity and size of the secondary PCR products by gel electrophoresis (Figure 1B). Then, the DIG-labeled SST probe was synthesized by *in vitro* transcription, which was preliminarily analyzed using gel electrophoresis (Figure 1C). The results show that the simplified method to prepare RNA probe was convenient and practicable.

### Key points for the stabilization of ISH signals

To identify the applicability of the probe, we detected the SST gene expression in the central lateral amygdala (CeL), which is known to be abundantly distributed with SST neurons (Li et al., 2013). The following parameters were then tested: proteinase K treatment and probe concentration.

Firstly, we examined the effect of proteinase K (PK), which was given to maximize penetration of the tissue (Jin and Lloyd, 1997; Newton et al., 2002; Ghafoory et al., 2012; Silahtaroglu, 2014). Sections were rinsed in 0.01 M PBST, proteinase K at room temperature (RT) and proteinase K at 37°C, respectively. The digesting condition of proteinase K was 2 μg/ml for 20 min (Jin and Lloyd, 1997; Newton et al., 2002). We found that the signal intensity of ISH treated with proteinase K at RT exhibited the strongest positive signals, compared with PBST and PK at 37°C (Figure 1D). Hence, the penetrating treatment with 2 μg/ml proteinase K at RT for 20 min was recommended in free-floating mouse brain sections with 40-μm thickness.

However, researchers have reported that the processing of proteinase K has its drawbacks in the attenuation of the immunofluorescence (IF) signal (Nehmé et al., 2011). 2 × SSC buffer, as a substitute, has been used to gain the stabilized ISH signals (Sonomura et al., 2008; Nehmé et al., 2011). To analyze the distinction of IF signals with penetration, we chose three treatments including 1 × PBST, 2 × SSCT, and 2 μg/ml PK, respectively. For the signal intensity of PKC-δ labeling with IF, the signal-noise ratio had little difference in the three groups. Comparing the signals of ISH, weaker density was observed in the 2 × SSCT group. Notably, the treatment of 2 μg/ml PK not only improved the signal-to-noise ratio of ISH but also resulted in a good signal of IF (Supplementary Figure 3).

Moreover, increasing concentrations of SST probe, 0, 0.5, 2, and 4 ng/μl were tested. The best specific vs. unspecific signal ratio was obtained at 0.5 and 2 ng/μl (Figure 1E). Therefore, the optimal concentrations of RNA probes were between 0.5 and 2 ng/μl in the free-floating brain sections of 40-μm thickness.

### Verification of three types of RNA probe

**Figure 2:**
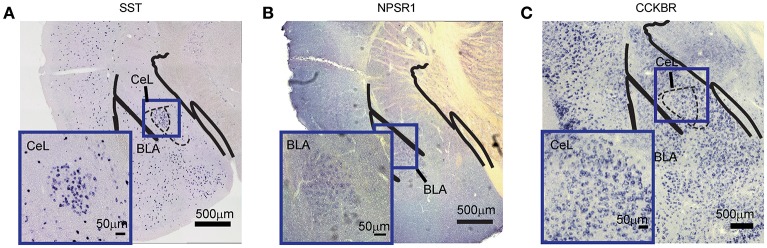
Detection of different mRNA species in mouse brain. **(A)** SST mRNA in the CeL. **(B)** NPSR1 mRNA in the BLA. **(C)** CCKBR mRNA in the CeL. The large images were captured with 10 × magnification (scale bar = 500 μm); the bottom-left images were captured with 20 × magnification (scale bar = 50 μm). NPSR1, neuropeptide S receptor 1; CCKBR, cholecystokinin B receptor; CeL, central lateral amygdala; BLA, basal lateral amygdala.

To validate the efficacy of this method for different mRNA transcripts, we chose three types of genes, namely somatostatin (SST), neuropeptide S receptor 1 (NPSR1), and cholecystokinin B receptor (CCKBR). SST is a peptide hormone regulating the endocrine system as well as affecting neurotransmission and cell proliferation (Gahete et al., 2010; Chanclón et al., 2012). NPSR1 is a member of the G-protein-coupled receptor binding neuropeptide S (NPS) and plays a role in arousal and anxiety-like behaviors (Neufang et al., 2015). CCKBR is a G protein-coupled receptor for gastrin and cholecystokinin (CCK), which primarily regulates anxiety, feeding, and locomotion in the brain (Ballaz et al., 2008). Using the primers listed in Table 1, we first prepared DIG-labeled anti-sense RNA probes. The DNA sequences were shown in Supplementary Figure 2. The hybridization conditions in this study were 2 ng/μl of each probe at 50°C for 16 h. The coloration time was different, which was related to the abundance of mRNA. The unambiguous and specific signals for those mRNAs in brain tissue were observed in Figures 2A–C.

### ISH applied in the transgenic reporter mouse

**Figure 3:**
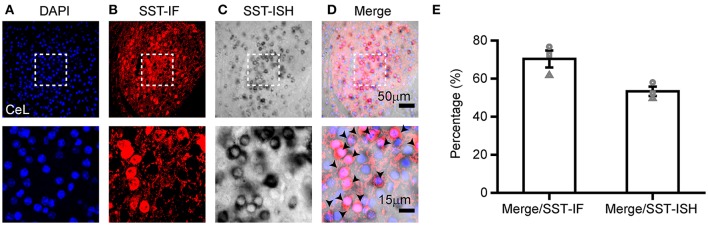
Application of ISH in transgenic reporter mice. SST expression in the CeL of the adult mouse brain that utilized **(A)** DAPI (blue), **(B)** immunofluorescence (IF) for protein(red), and **(C)** *in situ* hybridization for SST mRNA (black). **(D)** The co-localization of SST-IF, SST-ISH, and DAPI. Arrowheads indicate examples of neurons with colocalization; not all neurons with colocalization are indicated. **(E)** Quantification of the co-localization of SST-IF^+^ and SST-ISH^+^ (*n* = 3 mice). The data are presented as the mean ± S.E.M. DAPI, diamidino-phenylindole.

Mouse lines are widely used to visualize specific cell populations in the brain by transgenic expression with fluorescent proteins (Abe and Fujimori, 2013). Recent researchers have discovered that nerve cells are heterogeneous populations with different lifespans (Jackson and Alvarez-Buylla, 2008; Mirzadeh et al., 2008; Chojnacki and Weiss, 2009; Song et al., 2012; Giachino and Taylor, 2014; Semerci and Maletic-Savatic, 2016). To investigate the dynamics of SST neurons in the CeL of the adult mice, we tagged SST neurons by crossing the respective Cre driver mouse with a GFP reporter mouse (Ai3). The SST mRNA was detected by ISH, while SST-positive neurons labeled with GFP were observed by anti-GFP staining in the CeL (Figure 3A). 53 ± 2% of SST-positive neurons were GFP-positive neurons, most of which contained SST mRNA (70 ± 4%, *n* = 3) (Figure 3B). This result reveals that the SST probe realizes the recapitulative labeling of the GFP-tagged neurons, which contributes to providing an independent and sensitive assay for gene expression in the transgenic mouse.

### Combination of ISH and double immunohistochemistry

**Figure 4:**
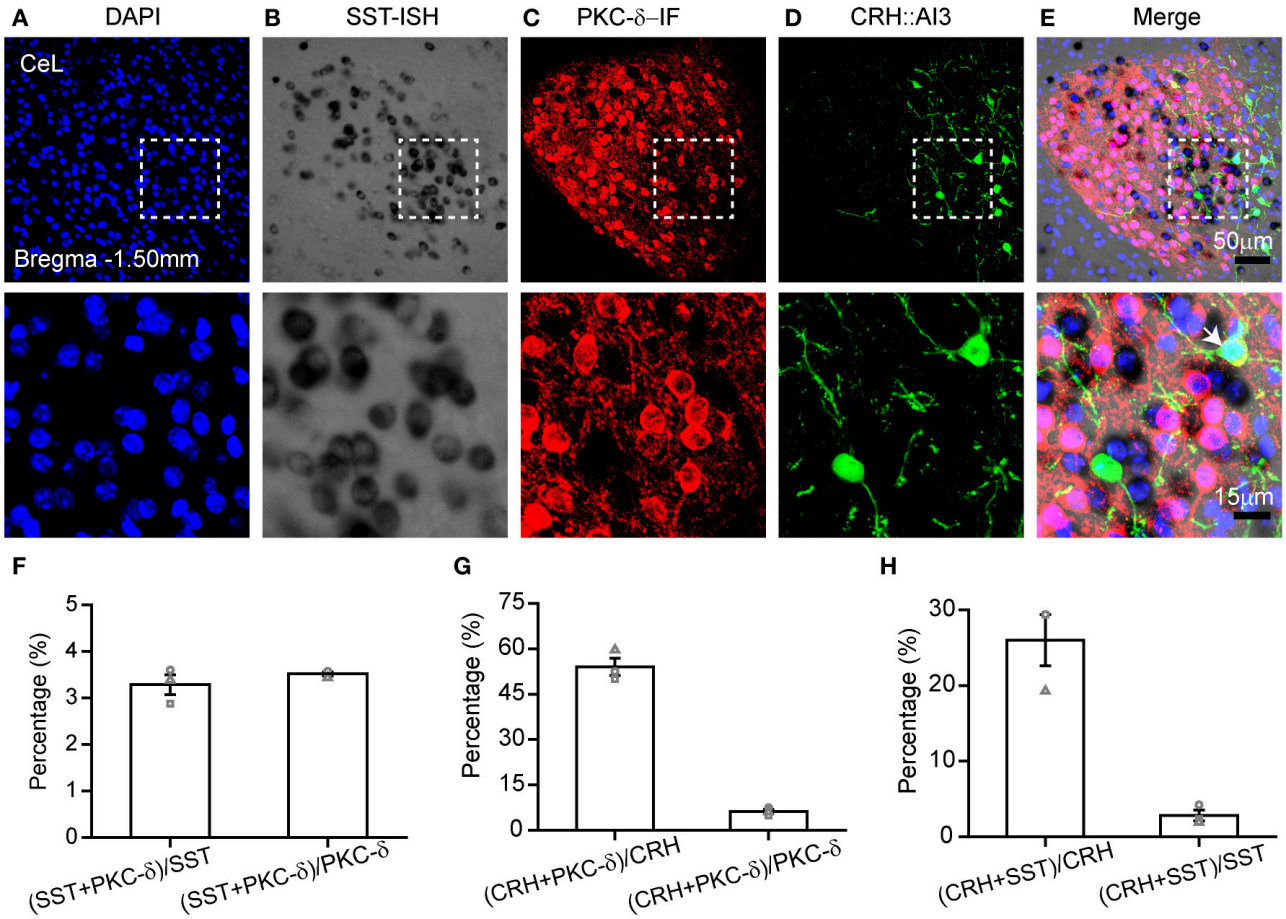
Combination of ISH with double immunofluorescence staining. Gene expression in the CeL that utilized **(A)** DAPI (blue), **(B)** *in situ* hybridization for SST mRNA (black), **(C)** immunofluorescence for PKC-δ protein (red), and **(D)** immunofluorescence for CRH protein (green). **(E)** The representative images for co-expression of SST, PKC-δ, CRH, and DAPI. The top images were obtained with 20 × magnification (scale bar = 50 μm), while the bottom images with 40 × magnification (scale bar = 15 μm). Arrowheads indicate examples of co-expressing neurons; not all co-expressing neurons are indicated. **(F)** Quantification of co-localization of SST^+^ and PKC-δ^+^. **(G)** Quantification of co-localization of PKC-δ^+^ and CRH^+^. **(H)** Quantification of co-localization of SST^+^ and CRH^+^. *n* = 3 mice. The data are presented as the mean ± S.E.M. PKC-δ, protein kinase C delta; CRH, adrenocorticotropic hormone.

ISH has been usually used to study co-expression with specific molecules combined with immunohistochemistry (Newton et al., 2002; Trifonov et al., 2009; Nehmé et al., 2011; Kudo et al., 2012; Parks et al., 2014). Previous studies show that the central lateral amygdala (CeL) contains three types of neurons expressing SST, PKC-δ, or CRH (Li et al., 2013; Wang et al., 2016). To investigate the co-localization of these pepdides/proteins, we tagged the CRH neurons by crossing the CRH-Cre mice with Ai3 mice. PKC-δ is labeled by immunofluorescence staining, and SST mRNA by ISH (Figures 4A–E). The fluorescence of GFP was extinct after hybridization, and thus GFP antibody was required for labeling CRH neurons. The results showed high specificity of labeling and low overlap (<5%) between SST and PKC-δ neurons in the CeL (Figure 4F). A total of 54 ± 3% of CRH neurons were PKC-δ-positive neurons, of which 6 ± 1% were CRH-positive (Figure 4G). A total of 26 ± 3% of CRH neurons expressed SST mRNA, little of which contained CRH (3 ± 1%) (Figure 4H). Thus, SST and PKC-δ neurons constitute a largely distinct populations in the CeL, as described previously (Li et al., 2013; Cui et al., 2017). In addition, most of the CRH neurons overlap with PKC-δ and SST neurons.

### Combination of ISH and retrograde tracing techniques

**Figure 5:**
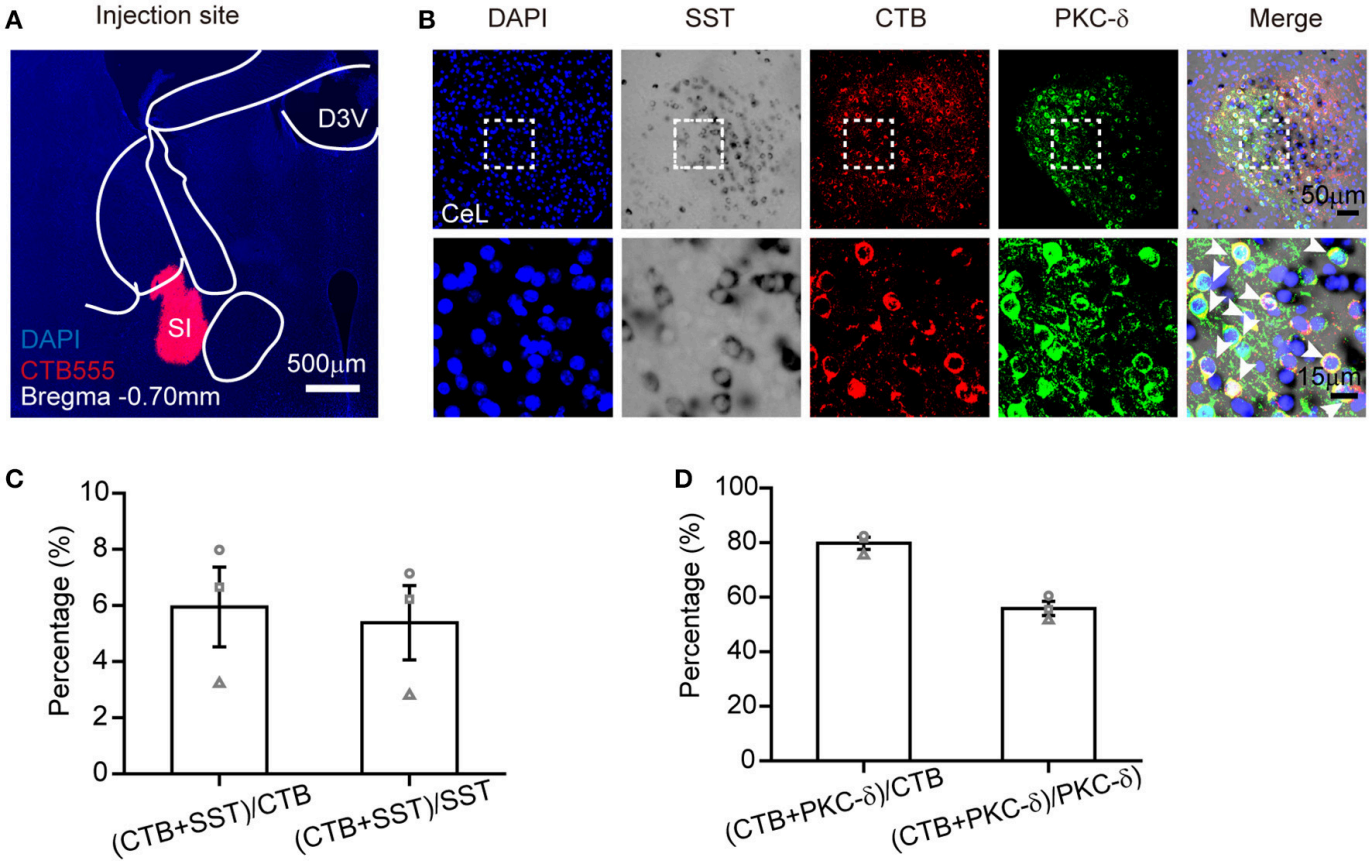
ISH combined with retrograde tracer labeling to visualize the functional connection between CeL and SI. **(A)** Injection site of Alexa Fluor 555-CTB (red) in the SI. **(B)** The representative images of retrogradely labeled CeL neurons projecting to the SI, containing immunofluorescence staining for CTB (red), *in situ* hybridization for SST mRNA (black), immunofluorescence staining for PKC-δ protein (green), and DAPI (blue). The upper images were obtained with 20 × magnification (scale bar = 50 μm), while the bottom images with 40 × magnification (scale bar = 15 μm). Arrowheads indicate examples of neurons with colocalization; not all neurons with colocalization are indicated. **(C)** Quantification of co-localization of CTB^+^ and SST^+^. **(D)** Quantification of co-localization of CTB^+^ and PKC-δ^+^. *n* = 3 mice. The data are presented as the mean ± S.E.M. SI, substantia innominate.

The combination of ISH with retrograde tracing can offer important neural connection information, as mentioned in previous reports (Le Moine, 2003; Conte-Perales et al., 2010). To verify the practicability of SST probe in retrograde tracing, we injected Alexa 555–CTB into the SI (Figure 5A) to investigate the co-expression of SST+, PKC-δ+, and CTB+ neurons in the CeL. The retrograde tracer was observed by the antibody of CTB due to the fluorescence extinction of Alexa 555–CTB after hybridization (Figure 5B). Using the combination of retrograde labeling and ISH methods, we demonstrated that 5 ± 1% of SST-positive neurons overlap with CTB-positive neurons. SST mRNA had little overlap (6 ± 1%) with CTB-positive neurons Figure 5C). Meanwhile, a total of 80 ± 2% of the CTB-positive neurons contained PKC-δ, which made up 56 ± 3% of CTB-positive neurons Figure 5D). These results reveal that most retrograde labeled CeL neurons expressed PKC-δ protein, but not SST mRNA, which was consistent with previous research (Cui et al., 2017).

## Discussion and conclusions

In the present study, we introduce a simplified protocol to prepare DIG-labeled RNA probes based on PCR amplification, and its various applications in neuroscience. We use highly sensitive DIG-labeled RNA probes, which can detect a variety of mRNA expression combined with alkaline phosphatase detection systems, constantly utilized in previous studies (Basyuk et al., 2000; Barroso-Chinea et al., 2007). Emphasis has been placed on the feasibility and usefulness of this method.

At the step of probe preparation, we make use of two pairs of primers to ensure the specificity of DNA sequences. The first pair of primer is used to obtain the specific DNA sequence from cDNA, while the secondary pair to add T7 promoter. A concentration of 50 ng/μl should be achieved for the first PCR, while 500 ng/μl is essential for the second PCR to ensure sufficient amount of template for *in vitro* transcription. The purified products of the secondary PCR are used directly for the synthesis of RNA probes with high efficiency. This method is timesaving and advantageous for rapid gene mapping.

At the ISH step, we summarize several key points for stable ISH signal and successful combination with IF (Supplementary Figure 4). (1) The treatment of tissue sections to enhance probe access to target mRNA is a critical step in the principle of ISH. The types of sections usually are frozen sections, paraffin sections and vibration sections. Frozen sections are highly susceptible to damage, which often leads to substantial loss of tissue architecture. Paraffin embedding method may reduce preservation of tissue mRNA level. In this study, we use the free-floating sections cut by vibratome to obtain good preservation of mRNA. (2) Preservation of target mRNA within tissues requires the use of crosslinking fixatives, such as formalin. Postfixation with 4% paraformaldehyde (PFA) can preserve the mRNA and morphology of the tissue, but excessive handing can decrease the combined capacity of probes (Jin and Lloyd, 1997; Yang et al., 1999; Nehmé et al., 2011; Silahtaroglu, 2014). Therefore, postfixation with 4% PFA for 6–8 h provides better probe access to the mouse brain. (3) Proteinase K is usually used to increase the penetration of the cell membrane, but insufficient or excessive digestion may result in unsatisfactory amplification and could induce false negatives, high background, or morphological abnormalities. In some cases, the probe can obtain sufficient ISH signal without any permeabilization (Wahle and Beckh, 1992). To obtain the optimized signal, the time and concentration of penetration needs to be determined by repeated experiments. (4) Certain RNase-free treatments are essential, especially for low-expressing mRNA, although some researchers have reported that a non-radioactive ISH method does not require RNase-free conditions (Tongiorgi et al., 1998). (5) Temperature of hybridization and post-hybridization treatment are important. Lower stringency can result in nonspecific binding, whereas higher stringency may result in a lack of signal. On the basis of the previous studies (Jin and Lloyd, 1997; Yang et al., 1999; Bonn et al., 2012) and our experiments, the recommended hybridization temperature is between 45° and 60°C in brain sections with 40-μm thickness. (6) The probe concentration is also vital for specific signal. Therefore, the actual conditions must be adjusted according to the tissue type and length of probes, which are conductive to detect various genes successfully.

In our study, we investigate the expression of SST mRNA in transgenic mice expressing GFP protein. However, we found that the co-localization of IF and ISH signals of GFP and SST was 70% in Figure 3, which may be due to the restrictions of transgenic strategy and the specificity of in situ. The transgenic strategies, including BAC (bacterial artificial chromosome) transgenic (Gong et al., 2003) and gene knock-in (Huang et al., 2014), have been widely used to generate transgenic mouse lines. Yet, these two strategies have certain limitations, such as the possibility of altering the expression of the targeted gene and incomplete expression of the endogenous gene. The transgene and endogenous gene can be detected by ISH. Therefore, ISH is an available method to confirm the reliability of the transgenic mouse line (Taniguchi et al., 2011).

A variety of ISH techniques, such as single-molecule FISH, double or triple-FISH, tyramide signal amplification (TSA)-ISH and in situ ISH, have been developed (Höltke and Kessler, 1990; Denkers et al., 2004; Campbell and Bartlett, 2007; Itzkovitz and van Oudenaarden, 2011; Lee et al., 2015). Fluorescent ISH (FISH) is a widely used method to detect nucleic acid sequences by fluorescently labeled probes (Itzkovitz and van Oudenaarden, 2011; Silahtaroglu, 2014), most of which are oligonucleotide probes (Moter and Gobel, 2000; Lécuyer et al., 2008). Because the traditional long probes are poorly permeant and have high background and low sensitivity (Itzkovitz and van Oudenaarden, 2011), the oligonucleotide probes of FISH can achieve broader applications. FISH is not only often used for finding specific features in DNA for use in genetic counseling, medicine, and species identification, but also to detect and localize specific RNA targets in cells and tissue samples (Matsuda and Chapman, 1995; Amann and Fuchs, 2008). Compared with FISH, the advantages of this method include: (1) simple and effective synthesis of RNA probe to identify the structure at the cellular level; (2) simplicity in the operation and low-cost for rapid gene mapping in neuroscience; (3) Convenient to control the intensity of ISH signals during the developing process; (4) Good compatibility with several other neuroanatomical techniques. The main drawback of this approach is the fact that it is not appropriate for investigations of subcellular structures and quantitative analysis.

The present method is applicable to a wide variety of molecules and has broad applications in neuroscience. There are still several problems to be overcome with repeated trials. Firstly, immediate-early gene (IEG), a marker of neural activity, has not been validated in our studies. Researchers have developed tyramide amplified immunohistochemistry–fluorescence ISH (TAI-FISH) to detect the IEG gene by multiple stimuli, which can label c-Fos protein successfully using TSA (Zhang et al., 2017). We will make an effort to combine ISH with IEG detection. Secondly, the probes will be applied in the virus tracing research to broaden the scope of application. Thirdly, the probes are encouraged to be used in combination with TSA system, which has been adopted as a means of enhancing signal strength to detect low-abundance mRNAs in Drosophila embryo and chromosome research. (Nehmé et al., 2011; Kudo et al., 2012; Silahtaroglu, 2014; Lee et al., 2015). After all, RNA probe based on the PCR method could be an appreciated tool and worthy of further research.

## Author contributions

HL: designed the research; RH: carried out experiments and analyzed results; SY: designed the primers and analyzed sequencing data; ML offered the technical support. All authors read and approved the final manuscript.

### Conflict of interest statement

The authors declare that the research was conducted in the absence of any commercial or financial relationships that could be construed as a potential conflict of interest. The reviewers WSun and WShen and the handling Editor declared their shared affiliation.

## Supplementary material

Supplementary Figure 1 Flow chart summarizing the preparation of RNA probes and applications in neuroscience. **(A)** Steps for preparing DIG-labeled RNA probes based on PCR amplification. **(B)** Steps for the application of ISH in transgenic mice. **(C)** Steps for the combination of ISH and double immunofluorescence staining. **(D)** Steps for the combined retrograde tracing and ISH.

Supplementary Figure 2 SST, NPSR1, and CCKBR sequences generated by PCR amplification.

Supplementary Figure 3 Evaluating three treatments for the immune-fluorescence signals of PKC-δ expression. In the CeL region of adult mouse brain, the IF signals of PKC-δ were examined after three treatments, including 1 × PBST **(A)**, 2 × SSCT **(B)**, and 2 μg/ml of PK **(C)**. The images were obtained with 20 × magnification (scale bar = 50 μm). *n* = 3 mice.

Supplementary Figure 4 Key points for good signals of ISH combined with IF.
