# Supplementary figures and images for: A PCR-Based Method for RNA Probes and Applications in Neuroscience
Source: Front Neurosci. 2018 May 2;12:266. doi: 10.3389/fnins.2018.00266 (PMC5942160; doi:10.3389/fnins.2018.00266)

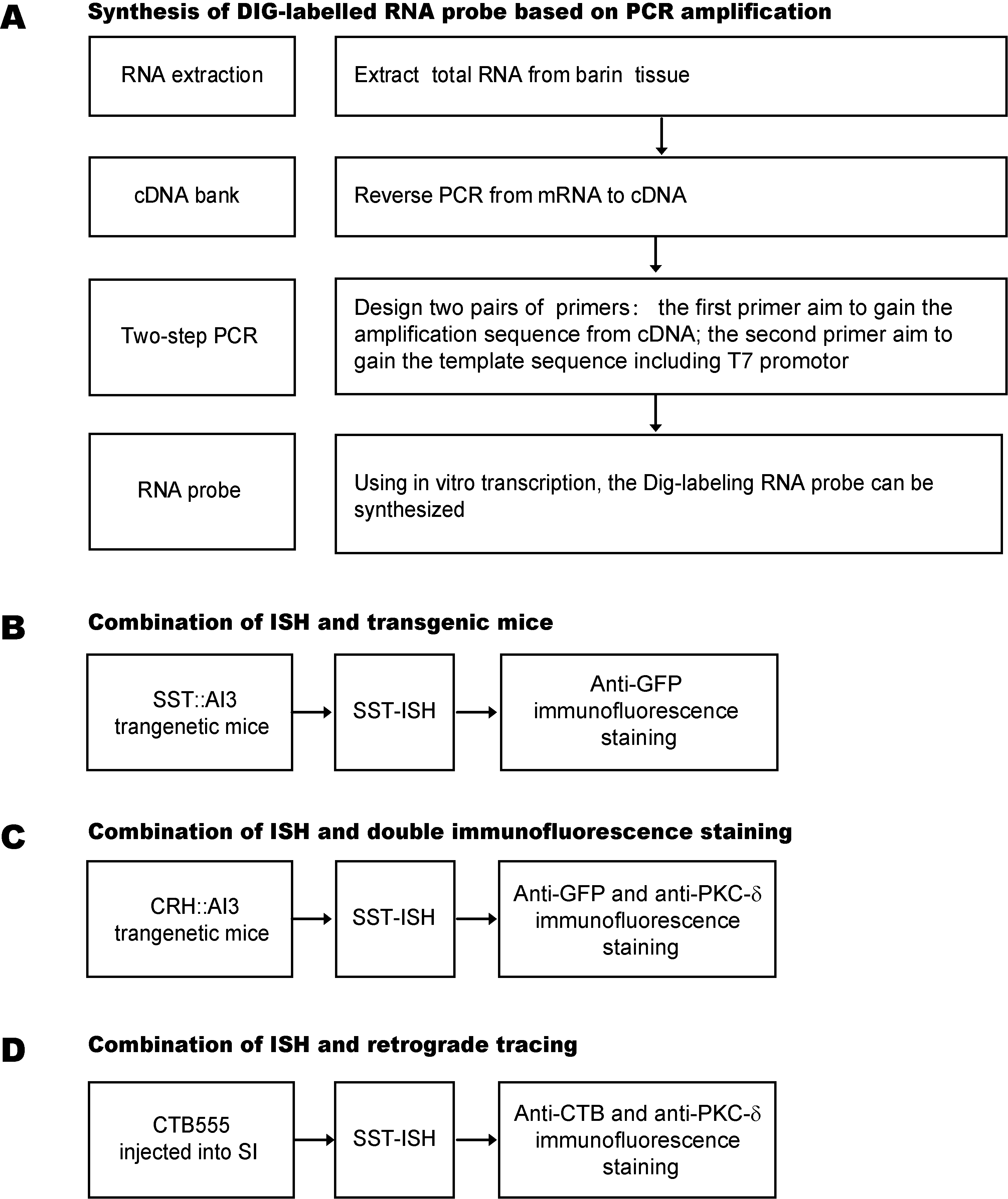

Supplement: Supplementary Figure 1 — Flow chart summarizing the preparation of RNA probes and applications in neuroscience. (A) Steps for preparing DIG-labeled RNA probes based on PCR amplification. (B) Steps for the application of ISH in transgenic mice. (C) Steps for the combination of ISH and double immunofluorescence staining. (D) Steps for the combined retrograde tracing and ISH. [file Image_1.TIF]

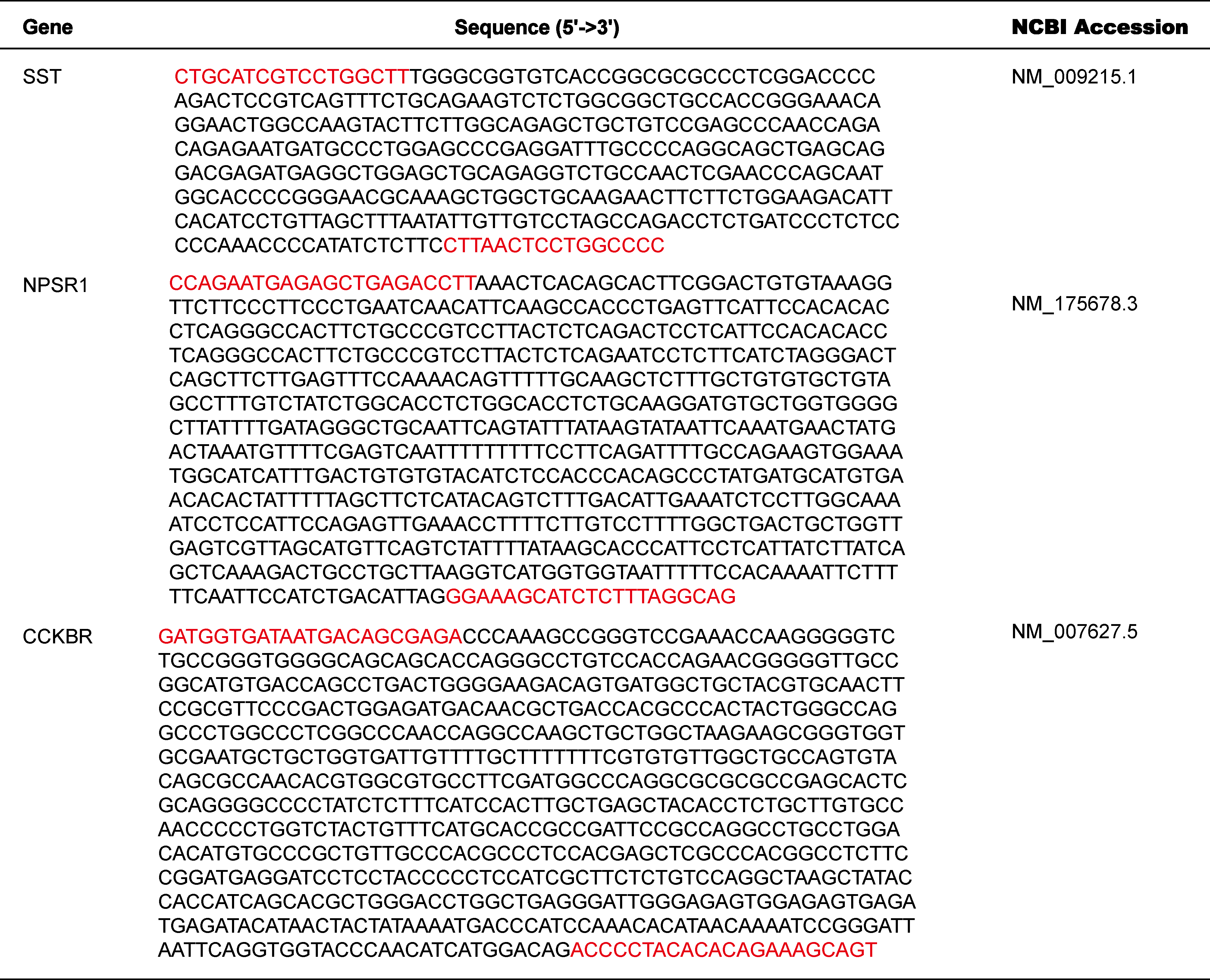

Supplement: Supplementary Figure 2 — SST, NPSR1, and CCKBR sequences generated by PCR amplification. [file Image_2.TIF]

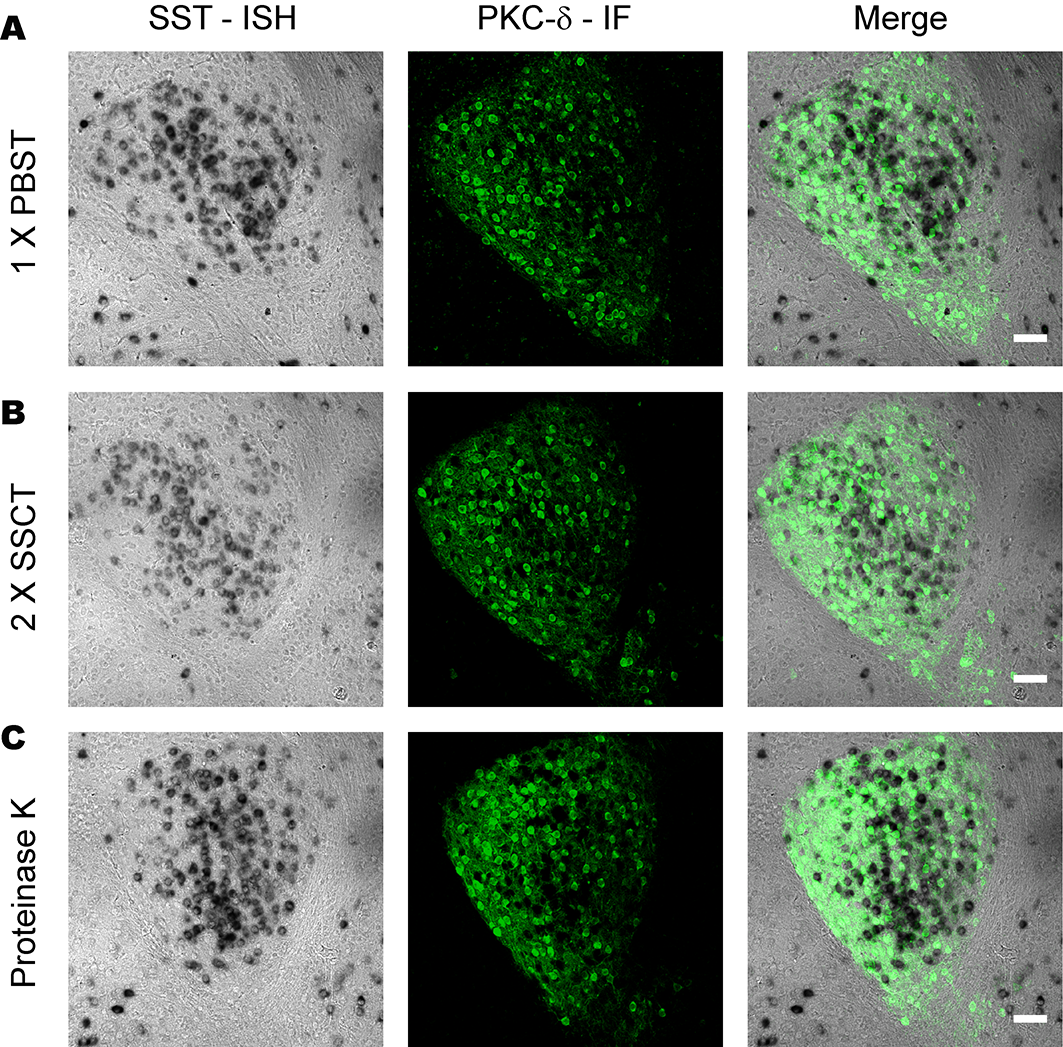

Supplement: Supplementary Figure 3 — Evaluating three treatments for the immune-fluorescence signals of PKC-δ expression. In the CeL region of adult mouse brain, the IF signals of PKC-δ were examined after three treatments, including 1 × PBST (A), 2 × SSCT (B), and 2 μg/ml of PK (C). The images were obtained with 20 × magnification (scale bar = 50 μm). n = 3 mice. [file Image_3.TIF]

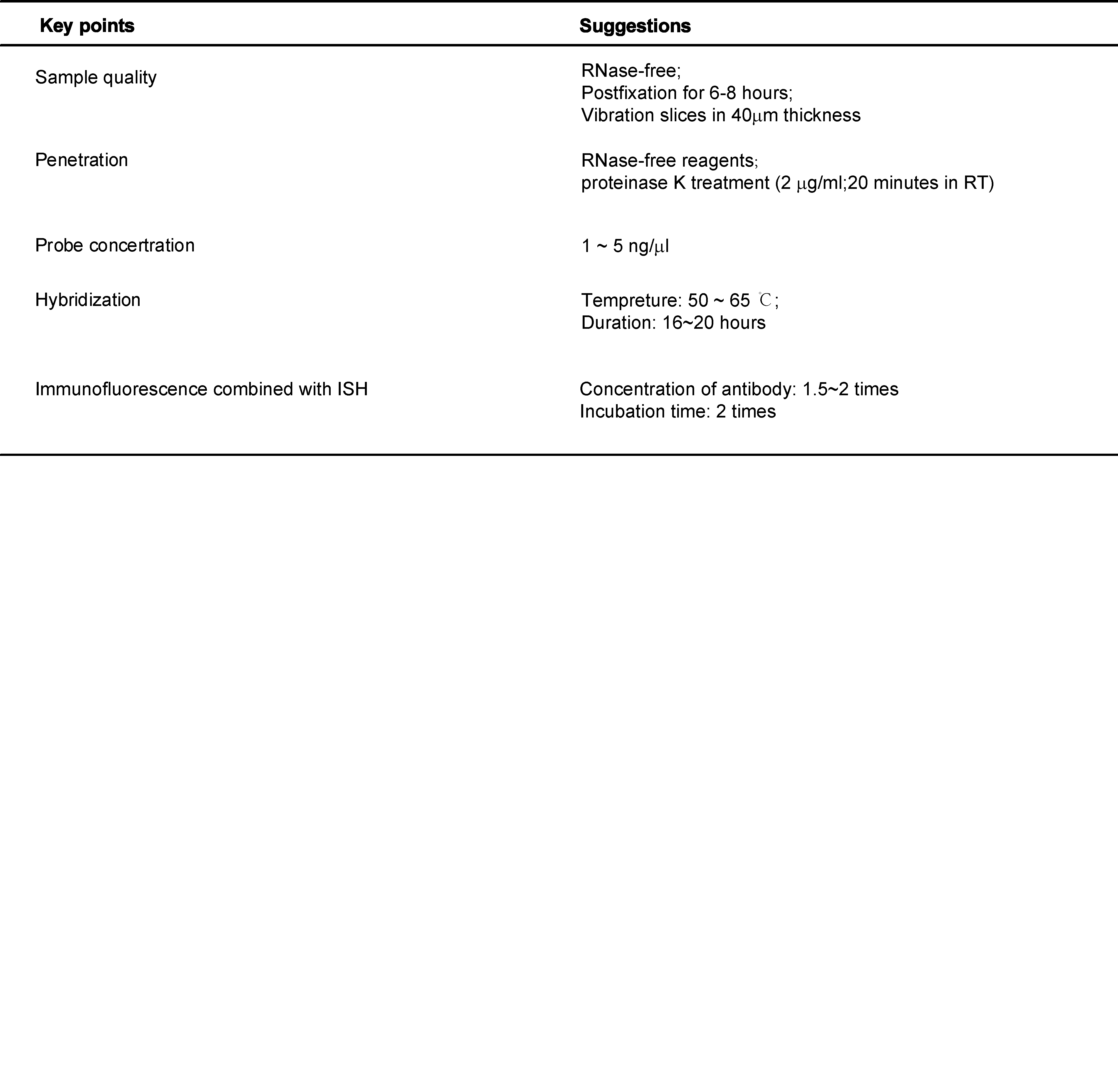

Supplement: Supplementary Figure 4 — Key points for good signals of ISH combined with IF. [file Image_4.TIF]
